# 392. 3D-DOSS – Using Digital Twins and Spatiotemporal Data Mapping for Infectious Disease Surveillance and Outbreak Investigations

**DOI:** 10.1093/ofid/ofab466.593

**Published:** 2021-12-04

**Authors:** Edwin Philip, Jean Xiang Ying Sim, Sean Whiteley, Andrew Hao Sen Fang, Weien Chow, Esther Lee, Jiunn Kee Lee, Low Seng Kee, Joseph Kin Meng Cheong, Shalvi Arora, Indumathi venkatachalam

**Affiliations:** 1 Singapore General Hospital, Singapore, Not Applicable, Singapore; 2 Axomem, Singapore, Not Applicable, Singapore; 3 SingHealth Polyclinics, Singapore, Not Applicable, Singapore; 4 Changi General Hospital, Singapore, Not Applicable, Singapore; 5 SingHealth, Singapore, Not Applicable, Singapore

## Abstract

**Background:**

The COVID-19 pandemic has brought to light the importance of contact tracing in outbreak management. Digital technologies have been leveraged to enhance contact tracing in community settings. However, within complex hospital environments, where patient and staff movement and interpersonal interactions are central to care delivery, tools for contact tracing and cluster detection remain limited. We aimed to develop a system to promptly, identify contacts in infectious disease exposures and detect infectious disease clusters.

**Methods:**

We prototyped a 3D mapping tool 3-Dimensional Disease Outbreak Surveillance System (3D-DOSS), to have a spatial representation of patients in the hospital inpatient locations. Based on the AutoCAD drawings, the hospital physical spaces are built within a game-development software to obtain accurate digital replicas. This concept borrows from the way gamers interact with the virtual world/space, to mimic the interactions in physical space, like the SIMS franchise. Clinical, laboratory and patient movement data is then integrated into the virtual map to develop syndromic and disease surveillance systems. Risk assignment to individuals exposed is through mathematical modeling based on distance coordinates, room type and ventilation parameters and whether the disease is transmitted via contact, droplet or airborne route.

**Results:**

We have mapped acute respiratory illness (ARI) data for the period September to December 2018. We identified an influenza cluster of 10 patients in November 2018. In a COVID-19 exposure involving a healthcare worker (HCW), we identified 44 primary and 162 secondary contacts who were then managed as per our standard exposure management protocols. MDRO outbreaks could also be mapped.

**Conclusion:**

Through early identification of at-risk contacts and detection of infectious disease clusters, the system can potentially facilitate interventions to prevent onward transmission. The system can also support security, environmental cleaning, bed assignment and other operational processes. Simulations of novel diseases outbreaks can enhance preparedness planning as health systems that had been better prepared have been more resilient in this current pandemic.

**Disclosures:**

**All Authors**: No reported disclosures

